# Correction: Rational design of a peptide capture agent for CXCL8 based on a model of the CXCL8:CXCR1 complex

**DOI:** 10.1039/c8ra90035c

**Published:** 2018-05-09

**Authors:** Dorothea Helmer, Ina Rink, James A. R. Dalton, Kevin Brahm, Marina Jöst, Tobias M. Nargang, Witali Blum, Parvesh Wadhwani, Gerald Brenner-Weiss, Bastian E. Rapp, Jesús Giraldo, Katja Schmitz

**Affiliations:** Technische Universität Darmstadt, Clemens-Schöpf-Institut Alarich-Weiss-Straße 4 64287 Darmstadt Germany schmitz@biochemie.tu-darmstadt.de +49 6151 16 72058 +49 6151 16 6964; Karlsruhe Institute of Technology, Institute of Microstructure Technology Hermann-von-Helmholtz-Platz 1 76344 Eggenstein-Leopoldshafen Germany; Laboratory of Molecular Neuropharmacology and Bioinformatics, Institut de Neurociències and Unitat de Bioestadística, Universitat Autònoma de Barcelona 08193 Bellaterra Spain; Karlsruhe Institute of Technology, Institute of Biological Interfaces (IBG-2) P.O. Box 3640 76021 Karlsruhe Germany; Karlsruhe Institute of Technology, Institute of Functional Interfaces Hermann-von-Helmholtz-Platz 1 76344 Eggenstein-Leopoldshafen Germany

## Abstract

Correction for ‘Rational design of a peptide capture agent for CXCL8 based on a model of the CXCL8:CXCR1 complex’ by Dorothea Helmer *et al.*, *RSC Adv.*, 2015, **5**, 25657–25668.

The authors regret that the original article included some results which were subsequently found to be based on a slightly different peptide sequence than the sequence originally reported. This issue is addressed in the following text, which is an update to the original article.

Upon further investigation of the IL8 capture peptide IL8-RP-Loops introduced in the original article, we found that during synthesis of the intended peptide sequence AKWRMVLRI–Ahx–ADTLMRTQ we had obtained the peptide AKWRMVLRI–Ahx–ADTLMRTE, in which the C-terminal glutamine was replaced with glutamic acid. This was confirmed by high resolution mass spectrometry. The reported high affinity (0.5 ± 0.3 μM) was reproduced by the E-mutant within experimental error (1.1 ± 0.1 μM) but not for the original sequence ending with glutamine. We conclude that all experiments were performed with the peptide AKWRMVLRI–Ahx–ADTLMRTE.

The affected amino acid Q271 was shown to be non-essential for receptor function by Hébert *et al.*^1^ So no essential amino acid of the original sequence was omitted in the exchange.

In the original publication we claimed that preorganization of the peptide is responsible for the high affinity of the receptor-derived peptide, as CD-spectroscopy had revealed a helical structure for the synthesized peptide. To test the effect of the exchange of glutamine with the structurally closely related glutamic acid on the preorganization of the peptide in solution, we ran a new set of molecular dynamics simulations on the peptide containing the original receptor sequence and the actually synthesized peptide IL8-RP-Loops. The helical content of the peptide structure over the course of the simulation was higher for IL8-RP-Loops with C-terminal glutamic acid (23.6%) than for the peptide ending with glutamine (6.0%). Thus, the serendipitous exchange of the polar glutamine at the C-terminus with negatively charged glutamic acid enhanced preorganization and led to the capture peptide with high affinity.

In conclusion, a capture peptide could be designed based on a binding region identified from a computational model of the CXCL8:CXCR1 complex. The peptide comprises receptor residues known to form essential contacts with the chemokine ligand and the exchange of the non-essential C-terminal glutamine for glutamic acid enhances the preorganization into a partially helical structure in solution responsible for its high affinity to the chemokine. In the design of receptor-derived capture peptides it is therefore important to examine the propensity to form secondary structure elements in solution to obtain high affinity peptide mimetics.

## Methods

Peptides were synthesized, purified and tested in fluorescence anisotropy binding assays as described in the original publication. High resolution mass spectra were obtained by MALDI-TOF MS on a 4800 Plus MALDI TOF/TOF Analyzer from AB SCIEX.

The structures of the IL8-RP-Loops derivatives were modeled using MODELLER.^2^ All simulations were performed in Gromacs^3^ using the Gromos65atb force field with parameters for the synthetic 6-amino-hexanoic acid linker.^4^ The simulation was run with an integration step size of 2 fs, and a van der Waals interaction cutoff value of 1.0 nm. The simulation temperature was set to 300 K and the water density was set to *ρ* = 1 for all studied peptides. The simulation was executed for a duration of 100 ns of explicit united-atom MD simulations. Define Secondary Structure of Proteins (DSSP)^5^ was computed for every frame and the mean occurrence of loops helical content 〈H〉 for the complete simulation was computed in R and bio3d.^6,7^

The Royal Society of Chemistry apologises for these errors and any consequent inconvenience to authors and readers.

## Supplementary Material
